# Die TSS in der Versorgung von Menschen mit psychischen Erkrankungen – Ergebnisse einer Psychotherapeutenbefragung

**DOI:** 10.1055/a-2797-1205

**Published:** 2026-04-22

**Authors:** Silke Neusser, Luisa Friedrich, Klemens Höfer, Sarah Schlierenkamp, Sandra Werner, Pauline Schlesiger, Dieter Best, Barbara Lubisch, Gebhard Hentschel, Franziska Weigel, Dagmar Wieczorek, Ursula Marschall, Christa Schaff, Bettina Meisel, Helene Timmermann, Anke Walendzik, Jürgen Wasem, Carina Abels

**Affiliations:** 1 Essener Forschungsinstitut für Medizinmanagement (EsFoMed) GmbH, Essen, Deutschland; 2 Lehrstuhl für Medizinmanagement, Universität Duisburg-Essen, Essen, Deutschland; 3 Deutsche Psychotherapeutenvereinigung (DPtV), Berlin, Deutschland; 4 AOK Bundesverband, Berlin, Deutschland; 5 BARMER, Wuppertal, Deutschland; 6 Berufsverband für Kinder- und Jugendpsychiatrie, Psychosomatik und Psychotherapie in Deutschland e. V. (bkjpp), Mainz, Deutschland; 7 Vereinigung für analytische und tiefenpsychologisch fundierte Kinder- und Jugendlichen-Psychotherapie in Deutschland e.V. gegr. 1953 (VAKJP), Berlin, Deutschland

**Keywords:** ambulante Versorgung, Psychotherapie, Reform der Psychotherapie-Richtlinie, Terminservicestelle, TSS, outpatient care, psychotherapy, reform of the psychotherapeutic directive, Appointment Service Centre, primary care

## Abstract

**Ziel:**

Die Terminservicestelle (TSS) wurde eingeführt, um Patienten den Zugang in die medizinische Versorgung sowie eine kurzfristige Terminkoordination zu erleichtern, insbesondere in der Psychotherapie.

**Methodik:**

Es wurden Psychotherapeuten in der vertragspsychotherapeutischen Versorgung von Erwachsenen befragt. Neben deskriptiven Analysen und Subgruppenanalysen wurden auch Korrelationsmaße und Effektgrößen ermittelt.

**Ergebnisse:**

Ein Drittel der 447 befragten Psychotherapeuten erkannte eine Verkürzung der Wartezeit bis zu einem Erstgespräch und 25,0% sahen die TSS als gut funktionierende zentrale Anlaufstelle. Als Hürde bei der Terminmeldung an die TSS wurde die fehlende Verfügbarkeit freier Kapazitäten gesehen.

**Schlussfolgerung:**

Die Ergebnisse verdeutlichen das Potenzial der TSS, den Zugang in die psychotherapeutische Versorgung zu verbessern, deuten aber auch auf zu überwindende Herausforderungen hin. Insbesondere Maßnahmen, die zu einer höheren Terminverfügbarkeit führen, könnten wirkungsvoll sein.

## Abstract (en)

**Objective:**

The appointment service centre (TSS) was introduced to facilitate patients’ access to medical care and to coordinate appointments at short notice, particularly in psychotherapy.

**Method:**

Psychotherapists providing contract-based psychotherapy to adults were surveyed. In addition to descriptive analyses and subgroup analyses, correlation measures and effect sizes were also determined.

**Results:**

One third of the 447 psychotherapists surveyed recognised a reduction in the waiting time for an initial consultation, and 25.0% considered the TSS to be a well-functioning central point of contact. The lack of available capacity was seen as an obstacle to making appointments with the TSS.

**Conclusion:**

The results highlight the potential of TSS to improve access to psychotherapeutic care, but also point to challenges that need to be overcome. Measures that lead to greater appointment availability could be particularly effective.

## Hintergrund

Die Terminservicestelle (TSS) wurde mit dem GKV-Versorgungsstärkungsgesetz 2015 im SGB V verankert und durch das Terminservice- und Versorgungsgesetz (TSVG) 1 weiter konkretisiert. Neben einem verbesserten, einfacheren und schnelleren Zugang der Patienten in die Versorgung sollte sich auch der Aufwand für die Terminkoordination für Bürger verringern 2 . Die TSS ist deutschlandweit verfügbar, wird jedoch über die Kassenärztlichen Vereinigungen (KV) regional organisiert, was innerhalb des gesetzlichen Rahmens zu KV-individuellen Regelungen führt.

Die TSS vermittelt Termine für diverse Fachgruppen, 2024 waren es insgesamt 1.592.829 Terminvermittlungen 3 . Den größten Anteil mit 23,1% der Terminbuchungen machen Termine bei Psychotherapeuten aus, gefolgt von Radiologen mit 19,0% der Termine 3 . Im Rahmen der psychotherapeutischen Versorgung vermittelt die TSS Termine für die psychotherapeutische Sprechstunde, die psychotherapeutische Akutbehandlung und probatorische Sitzungen. Damit Termine für psychotherapeutische Akutbehandlungen oder probatorische Sitzungen vermittelt werden können, ist ein Dringlichkeits- bzw. Vermittlungscode auf dem Formular „Individuelle Patienteninformation zur ambulanten Psychotherapeutischen Sprechstunde“, dem PTV 11 notwendig. Im Anschluss an die Sprechstunde erhält der Patient bei festgestelltem zeitnah notwendigen Behandlungsbedarf mit dem Formular eine Art Befundbericht von den Sprechstundentherapeuten 4 , wenn die Weiterbehandlung nicht in der eigenen Praxis möglich ist. Von den vermittelten Terminen in der Psychotherapie waren 2024 86,6% Termine für psychotherapeutische Sprechstunden, 3,1% für probatorische Sitzungen und 1,4% für psychotherapeutische Akutbehandlungen 3 . 1

Bisher existieren nur wenige wissenschaftliche Publikationen in Bezug auf die TSS. Sobiech-Eruhimovic und Martin (2021) untersuchten, ob die TSS zu einer Wartezeitverkürzung bei ausgewählten Facharztgruppen (exkl. Psychotherapeuten) in der Region der KV Westfalen-Lippe geführt hat 5 . Aufgrund der geringen Fallzahl der TSS vermittelten Termine im Beobachtungszeitraum 2016 bis 2018 kommen die Forscher u. a. zu dem Ergebnis, dass sie keine Aussage zur Veränderung der Wartezeit treffen können. Engesser et al. (2023) zogen Daten aus 2019 heran und betrachteten konkret die psychotherapeutischen Termine 6 . Sie kommen zu dem Fazit, dass insbesondere bei der Akutbehandlung und probatorischen Sitzungen in einigen Regionen Defizite bei der Vermittlung bestehen. Holle und Moosig (2019) betrachteten den Zeitraum von 2016 bis 2018 aus Perspektive der KV Schleswig-Holstein und zeigten eine hohe Anzahl an Fehlzuweisungen und Patienten, die ihren Termin nicht wahrnehmen (No-Show-Patienten, 28,4%) 7 . Die KV Bremen (2023) konnte auf Basis von Daten des ersten halben Jahres 2022 No-Show-Raten von durchschnittlich 21,0% identifizieren (im psychotherapeutischen Bereich 18,0–21,0%) 8 .

Es wird deutlich, dass die bisherigen Untersuchungen zur TSS regelmäßig auf einer Datenbasis vor 2020, also vor Inkrafttreten des TSVG, beruhen oder die relevante Fachgruppe der Psychotherapeuten gar nicht oder nur am Rande untersuchten. Zu berücksichtigen ist, dass die TSS ihr Potenzial nur dann entfalten kann, wenn die Vertragsärzte und -psychotherapeuten entsprechende Termine in ausreichendem Maße melden. Dies erfordert, trotz gesetzlicher Verankerung in §75 Abs. 1a SGB V, insbesondere die Akzeptanz der Vertragspsychotherapeuten gegenüber der TSS 9 .

Da der Erfolg der TSS maßgeblich durch die gemeldeten Termine beeinflusst wird, ist es Ziel dieses Artikels, die Ansichten der Psychotherapeuten zur TSS zu erfassen und in einem nächsten Schritt mögliche Optimierungspotenziale in Bezug auf die TSS zu identifizieren, woraufhin Lösungsvorschläge ausgearbeitet werden. Hierfür wurden Psychotherapeuten hinsichtlich ihrer Einschätzung bezogen auf die TSS als Institution, aber auch bezogen auf die Zusammenarbeit befragt.

Die Befragung fand im Rahmen des Innovationsfonds-Projekts Eva PT-RL (Förderkennzeichen: 01VSF19006) zur Evaluation der Reform der Psychotherapie-Richtlinie (PT-RL), welches ein positives Votum der Ethik-Kommission der Universität Duisburg-Essen (Votums-Zeichen: 20–9792-BO) erhielt, statt. Hierbei wurde neben den Veränderungen durch die Reform der PT-RL ebenfalls verschiedene Aspekte zur TSS untersucht (vgl. 10 ). Sowohl die Neuerungen der PT-RL als auch die TSS hatten das Potenzial den Zugang zur ambulanten psychotherapeutischen Versorgung für Patienten zu erleichtern und Wartezeiten zu reduzieren.

## Methodik

Im Rahmen des Projekts wurden Befragungen durchgeführt. In einer von diesen wurden deutschlandweit Psychotherapeuten angeschrieben, die in der vertragsärztlichen bzw. -psychotherapeutischen Versorgung von Erwachsenen tätig sind.

Es wurde eine geschichtete Zufallsstichprobe von 1.700 Psychotherapeuten für Erwachsene aus dem Pool des Zielgruppen-Spezialisten Acxiom Deutschland GmbH gezogen. Dieser bot eine Gesamtstichprobe von 34.151 psychologischen Psychotherapeuten, 5.270 niedergelassenen Ärzten der Fachrichtung Psychiatrie (mit Zusatz-Bezeichnung Psychotherapie) und 3.253 niedergelassenen Ärzten der Fachrichtung Psychosomatische Medizin und Psychotherapie an. Vorgegeben wurde die geschichtete Anschrift von 340 ärztlichen Psychotherapeuten (darunter 170 Ärzte der Fachrichtung Psychiatrie und Psychotherapie, 85 Ärzte der Fachrichtung psychosomatische Medizin und Psychotherapie, 85 Ärzte der Fachrichtung Psychotherapie) und 1.360 psychologischen Psychotherapeuten.

Der postalische Versand der Erhebungsbögen erfolgte im Juli 2021, ergänzt durch einen Reminder im Oktober 2021 aufgrund des geringen Rücklaufs.

Zeitgleich zur Befragung der Psychotherapeuten für Erwachsene wurde eine äquivalente Befragung von Kinder- und Jugendlichenpsychotherapeuten durchgeführt. Sofern diese angaben, dass mindestens ein Drittel ihrer Patienten auch Erwachsene sind, wurden diese ebenfalls in die Auswertung der Fragebögen der Psychotherapeuten für Erwachsene einbezogen.

Der standardisierte Erhebungsbogen wurde auf Basis einer strukturierten Literaturrecherche und sechs vorangestellten Fokusgruppen sowie fünf Einzelinterviews 11 entwickelt und einem Pretest unterzogen. Der 24-seitige Erhebungsbogen umfasste neben fachlich-inhaltsbezogenen Angaben u. a. auch demografische Aspekte, den beruflichen Hintergrund, die Praxisform, Angaben zur versorgten Patientengruppe sowie der Region, in der die Praxis liegt. Abgefragt wurden neben soziodemografischen Angaben auch verschiedene Aspekte im Zusammenhang mit der Reform der PT-RL sowie entsprechend zur TSS. Dabei wurden verschiedene Fragetypen (halbgeschlossene Fragen, Ein- und Mehrfachantwortfragen, fünfstufige Likert-Skalen) genutzt. Der vollständige Ergebungsbogen kann in Anlage 4 des Ergebnisberichtes zum Projekt eingesehen werden 12 .

Die Teilnahme an der Befragung war sowohl online (über LimeSurvey im Rahmen des Reminders) als auch papierbasiert möglich. Die LimeSurvey Daten wurden in IBM SPSS Statistics 25 überführt und mit den Daten der eingescannten Papierbögen für die Auswertung zusammengeführt. Die Aufbereitung und deskriptive Auswertung erfolgte durch die EsFoMed GmbH.

Im Zuge der deskriptiven Statistik wurden absolute und relative Häufigkeiten berechnet. Zur Erfassung von Subgruppenunterschieden wurden die Variablen zur TSS mittels Chi²-Tests nach Alter, Qualifikation, Weiterbildung, KV-Region, Gemeindegröße und Praxistyp stratifiziert. Neben der statistischen Signifikanz wurde auch die Effektstärke mittels der Koeffizienten Phi, Cramer’s V und p-Werten erfasst. Signifikanz wird im Rahmen des Artikels bei einem Alpha von 0,05 angenommen (p<0,05).

## Ergebnisse

Es beteiligten sich 447 Psychotherapeuten an der Befragung. Der mehr als überwiegende Teil der Befragten sendete einen Papierfragebogen zurück. Lediglich 3,6% nahmen online via LimeSurvey teil. Von den teilnehmenden Psychotherapeuten waren mehr als zwei Drittel 51 Jahre oder älter und mehrheitlich (77,2%) weiblich. Mit 89,2% beteiligten sich überwiegend psychologische Psychotherapeuten an der Befragung, die übrigen waren bis auf einen kleinen Teil ärztliche Psychotherapeuten. Mehr als die Hälfte der Befragten (56,6%) war für das Psychotherapieverfahren Verhaltenstherapie zugelassen. Ein Viertel (25,1%) nutzte tiefenpsychologische Therapie und weitere 12,9% analytisch und tiefenpsychologische Therapie ( Tab. 1 ).

**Table:** **Tab. 1** Merkmale der Psychotherapeuten.

Merkmal		n	(%)
** Altersgruppe ^1^ (n=443) **	21–40	42	(9,5)
	41–50	100	(22,6)
	51–60	155	(35,0)
	Über 60	146	(33,0)
**Geschlecht (n=429)**	Männlich	98	(22,8)
	Weiblich	331	(77,2)
**Absolvierter Studiengang (n=436)**	Medizin	41	(9,4)
	Psychologie	389	(89,2)
	Anderes Studium	6	(1,4)
**Psychotherapeutische Verfahren (n=436)**	Nur tiefenpsychologisch	111	(25,1)
	Nur Verhaltenstherapie	250	(56,6)
	Analytisch und tiefenpsychologisch	57	(12,9)
	Sonstige ^2^	24	(5,4)
**Größe des Ortes in dem praktiziert wird (n=441)**	Landgemeinde (<5.000 Einwohnende)	35	(7,9)
	Kleinstadt (5.000 bis<20.000 Einwohnende)	69	(15,6)
	Mittelstadt (20.000 bis 100.000 Einwohnende)	110	(24,9)
	Großstadt (ab 100.000 Einwohnende)	227	(51,5)
** KV Region ^3^ (n=435) **	KV Region West	156	(35,2)
	KV Region Nord	60	(13,5)
	KV Region Süd	125	(28,2)
	KV Region Ost	44	(9,9)
	Berlin	34	(7,7)
	Hamburg	24	(5,4)
**Behandelte Patientengruppe (n=441)**	Erwachsene	396	(89,9)
	Erwachsene sowie Kinder und Jugendliche ^4^	45	(10,1)

^1^ Aufgrund geringer Fallzahlen wurden die Altersgruppen 21–30 und 31–40 zusammengefasst;

^2^ Sonstige: Systemische Psychotherapeuten sowie sonstige Kombinationen der Verfahren; Niemand bot ausschließlich eine analytische Therapie an;

^3^ KV-Region West (Westfalen-Lippe, Nordrhein, Hessen, Rheinland-Pfalz, Saarland), KV-Region Nord (Schleswig-Holstein, Niedersachsen, Bremen), KV-Region Süd (Baden-Württemberg, Bayern), KV-Region Ost (Mecklenburg-Vorpommern, Brandenburg, Sachsen, Sachsen-Anhalt, Thüringen);

^4^ Wenn beide Gruppen mindestens einen Anteil von ca. einem Drittel der betreuten Patienten ausmacht.

Die befragten Psychotherapeuten beurteilten die Aussage, dass die TSS als zentrale Anlaufstelle gut funktioniere, mehrheitlich negativ. Lediglich 25,0% der Befragten stimmten der Aussage (voll) zu. Auch die Aussage, dass die TSS dazu führe, dass Patienten auf freie Plätze verteilt werden, wurde von 58,3% der Befragten verneint. Lediglich ein Drittel der Psychotherapeuten war der Meinung, dass die TSS dazu beigetragen habe, den Zeitraum bis zu einer psychotherapeutischen Sprechstunde zu verkürzen ( Tab. 2 , **Anhang 1** ). Bezogen auf diese drei geschilderten Aussagen, wurden lediglich bezogen auf die Anlaufstelle und Wartezeit Subgruppenunterschiede zwischen verschiedenen KV-Regionen und der Gemeindegröße deutlich ( **Anhang 3** ).

**Table:** **Tab. 2** Erfahrungen mit und Einschätzung zur TSS.

Erfahrungen mit und Einschätzung zur TSS	Stimme gar nicht zu n (%)	Stimme nicht zu n (%)	Teils, teils n (%)	Stimme zu n (%)	Stimme voll zu n (%)
**Beurteilung der Tätigkeit der TSS**					
Die TSS funktioniert gut als zentrale Anlaufstelle. (n=365)	80 (21,9)	85 (23,3)	109 (29,9)	71 (19,5)	20 (5,5)
Die TSS führt dazu, dass Patienten auf freie Plätze verteilt werden. (n=353)	101 (28,6)	105 (29,7)	83 (23,5)	52 (14,7)	12 (3,4)
Die TSS hat dazu beigetragen, dass die Wartezeiten für ein Erstgespräch (=psychotherapeutische Sprechstunde) verkürzt werden konnten. (n=365)	117 (32,1)	62 (17,0)	65 (17,8)	93 (25,5)	28 (7,7)
**Nutzung der gemeldeten Termine**					
Die von mir gemeldeten Termine wurden nicht genutzt und sind verfallen. (n=274)	46 (16,8)	45 (16,4)	116 (42,3)	34 (12,4)	33 (12,0)
Die von der TSS mir zugewiesenen Patienten haben häufig lange Anfahrtswege. (n=250)	14 (5,6)	64 (25,6)	111 (44,4)	37 (14,8)	24 (9,6)
Die von der TSS mir zugewiesenen Patienten kommen nicht zu den vereinbarten Terminen und führen zu Ausfallzeiten. (n=274)	18 (6,6)	49 (17,9)	136 (49,6)	39 (14,2)	32 (11,7)
**Terminmeldung durch die Psychotherapeuten**					
Ich habe keine Kapazitäten, um Termine an die TSS zu melden. (n=417)	34 (8,2)	49 (11,8)	61 (14,6)	75 (18,0)	198 (47,5)
Ich melde freie Termine für die psychotherapeutische Sprechstunde an die TSS. (n=294)	35 (11,9)	24 (8,2)	60 (20,4)	87 (29,6)	88 (29,9)
Ich melde freie Termine für die psychotherapeutische Akutbehandlung an die TSS. (n=286)	129 (45,1)	67 (23,4)	43 (15,0)	18 (6,3)	29 (10,1)
Ich melde freie Termine für probatorische Sitzungen an die TSS. (n=288)	138 (47,9)	57 (19,8)	35 (12,2)	30 (10,4)	28 (9,7)

Hinsichtlich der Aussage, dass die jeweils gemeldeten Termine nicht genutzt wurden und verfallen sind, zeigte sich ein heterogenes Meinungsbild (42,3% teils, teils, 33,2% stimmten nicht zu, 24,4% stimmten zu). Auch hier zeigten sich Subgruppenunterschiede bezogen auf die KV-Region ( **Anhang 4** ). Ähnlich heterogen war die Wahrnehmung des Problems, dass vergebene Termine von Patienten nicht wahrgenommen wurden und verfallen sind. Dieser Aussage stimmten 24,4% zu und 42,2% gaben teils, teils an ( Tab. 2 , **Anhang 1** ).

Bezogen auf das eigene Meldeverhalten wurde als grundsätzliche Herausforderung genannt, dass keine Kapazitäten bestehen würden, um Termine an die TSS zu melden. Dieser Aussage stimmten 65,5% (voll) zu, weitere 14,6% gaben teils, teils an. Betrachtet man die unterschiedlichen Therapiebausteine wird deutlich, dass, sofern Termine gemeldet wurden, diese mehrheitlich die psychotherapeutische Sprechstunde (58,5%) und deutlich seltener die psychotherapeutische Akutbehandlung (16,4%) oder Probatorik (21,1%) betrafen ( Tab. 2 , **Anhang 1** ). Bezogen auf das Meldeverhalten für psychotherapeutische Sprechstunden zeigten sich neben Unterschieden hinsichtlich der KV-Region auch solche bezogen auf das Geschlecht ( **Anhang 5** ).

Ergänzend wurden die Psychotherapeuten zu ihrer Zufriedenheit mit der Zusammenarbeit mit der TSS befragt. Insgesamt waren 50,3% der Psychotherapeuten mit der TSS-Zusammenarbeit eher bis sehr zufrieden. Des Weiteren wurden drei Teilaspekte der TSS betrachtet; die Informationen, die die TSS an Patienten vermittelt, die Terminvergabe und der zeitliche Aufwand, um Termine zu melden. Hier zeigten sich vergleichbare Ergebnisse bezüglich der Zufriedenheit, wobei die Informationsweitergabe an Patienten am schlechtesten bewertet wurde. Die Psychotherapeuten waren überwiegend geteilter Meinung (33,0% teils, teils) oder eher unzufrieden (27,7%) mit den Informationen, die die TSS an Patienten vermittelte ( Tab. 3 , **Anhang 2** ). Subgruppenunterschiede bei der Zufriedenheit zeigten sich auch dabei im Vergleich der KV-Regionen ( **Anhang 6** ).

**Table:** **Tab. 3** Beurteilung der Zusammenarbeit mit der TSS.

Beurteilung der Zufriedenheit …	Sehr unzufriedenn (%)	Eher unzufriedenn (%)	Teils, teils n (%)	Eher zufriedenn (%)	Sehr zufriedenn (%)
**Mit der Zusammenarbeit insgesamt (n=272)**	21 (7,7)	44 (16,2)	70 (25,7)	107 (39,3)	30 (11,0)
**Mit den Informationen, die die TSS an Patienten vermittelt (n=267)**	32 (12,0)	74 (27,7)	88 (33,0)	56 (21,0)	17 (6,4)
**Mit der Terminvergabe (n=267)**	21 (7,9)	37 (13,9)	58 (21,7)	113 (42,3)	38 (14,2)
**Mit dem zeitlichen Aufwand, um Termine zu melden (n=272)**	38 (14,0)	60 (22,1)	40 (14,7)	95 (34,9)	39 (14,3)

Die detaillierten Ergebnisse der Subgruppenanalysen können den Anhängen entnommen werden ( **Anhang 3 bis 6** ). Wie oben bereits dargestellt, gab es Subgruppenunterschiede überwiegend bezogen auf die KV-Region, in der der Psychotherapeut tätig war.

## Diskussion

Die TSS ist für die psychotherapeutische Versorgung im Vergleich zu anderen Fachgruppen von besonderer Bedeutung, da die meisten vergebenen Termine auf diesen Fachbereich entfallen 3 . Entsprechend zielt der Artikel darauf ab, die Befragungsergebnisse bezogen auf die Ansichten der Psychotherapeuten zur TSS strukturiert wiederzugeben und mögliche Optimierungspotenziale in Bezug auf die TSS zu identifizieren.

Die Befragung im Eva PT-RL Projekt zeigt, dass die TSS ihr intendiertes Ziel als zentrale Anlaufstelle eine Verkürzung der Zeit bis zu einem Erstkontakt zu ermöglichen nach Ansicht der Psychotherapeuten noch nicht erreicht hat. Als Hürde wurde, neben fehlenden zeitlichen Kapazitäten bei der Terminbereitstellung, u. a. der Anteil an Patienten, die nicht zu den vermittelten Terminen erscheinen, gesehen. Sofern Psychotherapeuten bereits mit der TSS zusammengearbeitet haben, wurde diese Zusammenarbeit als positiv eingeschätzt. In den Tab. 2 und 3 wird deutlich, dass sich bei einigen Aussagen ein heterogenes Stimmungsbild (z. B. gemeldete Termine wurden nicht genutzt) zeigte, während anderen mehrheitlich zugestimmt (z. B. keine Kapazitäten zur Meldung von Terminen an die TSS) oder diese mehrheitlich abgelehnt (z. B. Verteilung auf freie Plätze) wurden.

Im Jahr 2024 wurden von den Psychotherapeuten insgesamt 431.408 Terminbuchung zur Verfügung gestellt (2023: 366.421). Von diesen zur Verfügung gestellten Terminen wurden 81,0% (2023: 87,8%) vergeben. Entsprechend blieben 19,0% der eingestellten Termine ungenutzt. In der Eva PT-RL-Befragung stimmten 24,4% der Psychotherapeuten der Aussage (voll) zu, dass die von ihnen eingestellten Termine ungenutzt geblieben sind. Zusätzlich wurden 2024 12,8% (2023: 12,3%) der bereits gebuchten Termine wieder vom Patienten oder der Praxis abgesagt 3 , 13 . Der Anteil all jener Patienten, die einen gebuchten Termin nicht wahrgenommen hat, könnte in der Versorgungsrealität noch höher sein als die im TSS-Evaluationsbericht angegebenen 12,8%, da dort die Patienten, die den Termin ohne Abmeldung nicht wahrgenommen haben, nicht erfasst wurden. Diese Aussage wird gestützt durch die Analyse der KV Bremen (2023), in der im psychotherapeutischen Bereich No-Show-Raten zwischen 18,0% und 21,0% in Q1/Q2 2022 ermittelt wurden 8 . Im Rahmen der hier vorgestellten Befragung stimmte etwa jeder Vierte der Aussage zu, dass von der TSS zugewiesene Patienten nicht zu den vereinbarten Terminen gekommen sind, was zu Ausfallzeiten führte. Dies könnte dazu beitragen, dass Psychotherapeuten ihre Termine favorisiert ohne Beteiligung der TSS vergeben, obgleich es sich um einen niedrigschwelligen Zugangsweg für Patienten handelt. Es fehlt jedoch aktuell an Literatur, wie hoch die No-Show-Raten ohne Beteiligung der TSS sind.

Versorgungsbereichsübergreifend wurden 2024 1.592.829 (2023: 1.227.417) Termine vermittelt. Dem gegenüber stehen 2024 3.424.985 (2023: 2.211.781) Vermittlungswünsche. Im Bereich der Psychotherapie mündeten 2024 38,4% der Vermittlungswünsche in einem vermittelten Termin 3 , 13 . 2023 lag diese Quote noch bei 45,8%. Verknüpft mit den vorher genannten Ergebnissen, dass 81,0% der von Psychotherapeuten gemeldeten Termine vergeben wurden, folgt daraus einerseits, dass nicht alle gemeldeten Termine der Psychotherapeuten vermittelt werden können und andererseits deutlich weniger als die Hälfte der Vermittlungswünsche für psychotherapeutische Leistungen bedient werden können. Dies könnte ein Erklärungsansatz für die Einschätzungen der Psychotherapeuten in der Befragung des Eva PT-RL Projekts sein, in dem sowohl die TSS als zentrale Anlaufstelle als auch ihre Verteilung der Patienten auf freie Plätze als eher kritisch beurteilt wurde.

Damit die TSS ihr Potenzial zukünftig ausschöpfen kann, sollten die beschriebenen Herausforderungen adressiert werden. Mögliche Lösungsansätze könnten daher an verschiedenen Stellen ansetzen.

Um den Anteil der Patienten, der den von der TSS vermittelten Termin wahrnimmt, zu erhöhen, haben einige KVen die Verpflichtung eingeführt, dass Patienten die von der TSS vermittelten Termine in der Praxis zu bestätigen haben 14 . Eine weitere Maßnahme, die zu einer höheren Inanspruchnahme der gebuchten Termine beitragen könnte, ist eine automatisierte und standardisierte Erinnerung des Patienten durch die TSS. Der optimale Zeitpunkt für die Erinnerung sollte Gegenstand weiterführender Forschung sein. Dass sowohl telefonische Erinnerung, also auch Mobiltelefonnachrichten (z. B. SMS) einen positiven Einfluss auf die Inanspruchnahme von Terminen haben, wurde bereits in mehreren Studien gezeigt (z. B. 15 ). Die Erinnerung der TSS sollte neben dem genauen Zeitpunkt des vereinbarten Termins auch die Praxis und ihren Standort angeben. Aktuell bestehende rechtliche und organisatorische Hürden, die etwa in Datenschutz-Einschränkungen liegen, müssten abgebaut werden.

Eine weitere Herausforderung liegt in der Terminallokation. Um diese zu verbessern, wird eine solide Datengrundlage benötigt. Auf KV-Ebene sollten Erhebungen durchgeführt werden, welche Therapieelemente in welcher Frequenz und Menge nachgefragt werden (psychotherapeutische Sprechstunde, Probatorik, psychotherapeutische Akutbehandlung). Abhängig hiervon könnten KVen konkrete und an der tatsächlichen Nachfrage orientierte Empfehlungen an ihre Vertragspsychotherapeuten herausgeben, wie viele Termine von welchem Therapieelement benötigt werden. Eine solche Empfehlung kann Psychotherapeuten als Orientierung bei der Terminmeldung dienen und zu einem verstärkten Meldeverhalten bei gleichzeitig verbesserten Allokationschancen beitragen. In diesem Zusammenhang kann auch der unterschiedlichen Einbindung der Therapieelemente im Versorgungsprozess Rechnung getragen werden (z. B. einer regelhaft folgenden Richtlinien-Therapie im Anschluss an probatorische Sitzungen). Eine verbesserte Terminallokation könnte auch dazu führen, dass die Anfahrtswege für Patienten verringert werden. 2024 wurden diese bei 24 Minuten mit dem PKW bzw. 16 Kilometern angegeben. In dieser Berechnung sind jedoch die Absagen bereits exkludiert. Hiermit liegen die Psychotherapeuten deutlich innerhalb der Grenzen der zumutbaren Entfernung 3 . Die Psychotherapeuten in der hier vorgestellten Befragung zeigten ein heterogenes Bild hinsichtlich der Einschätzung, ob die durch die TSS vermittelten Patienten häufig lange Anfahrtswege haben.

Insgesamt sollte das Potenzial der TSS als zentrale Anlaufstelle für die Vereinbarung von Facharzt- und Psychotherapeutenterminen und zur Wartezeitreduktion weiter untersucht werden. Besonders relevant hierbei erscheint die Akzeptanz der Leistungserbringer und wie diese verbessert werden kann. Zusätzlich müssen weitere Faktoren, die sich bspw. auf das Meldeverhalten der Psychotherapeuten auswirken (z. B. Bedarfsplanung) berücksichtigt werden, standen aber hier nicht im Fokus.

Die Erfassung der Perspektive der Psychotherapeuten geschah auf Basis einer standardisierten Querschnittsbefragung. Daher besteht die grundsätzliche Einschränkung von nicht-experimentellen Querschnittsstudien, dass keine Schlussfolgerungen zur Kausalität möglich sind. Für die Befragung wurde aus dem Pool eines Zielgruppen-Spezialisten eine Zufallsstichprobe gezogen und angeschrieben. Allerdings deckte dieser Adresspool nicht alle Psychotherapeuten mit KV-Zulassung ab. Ein Abgleich mit den Daten der KBV für das Jahr 2021 zeigt eine Annäherung der Befragungsteilnehmenden im Eva PT-RL Projekt an die Grundgesamtheit. Auf Bundesebene waren 66,4% der ärztliche Psychotherapeuten und 76,3% der psychologischen Psychotherapeuten in der vertragsärztlichen Versorgung weiblich 16 . Über 51 Jahre alt waren 89,2% der ärztlichen Psychotherapeuten und 57,6% der psychologischen Psychotherapeuten 17 . Auch die Befragungsverteilung hinsichtlich KV-Zugehörigkeit nähert sich den realen Anteilen an. 2021 waren 33,6% der ärztlichen Psychotherapeuten in der KV-Region West, 11,4% in der Region Nord, 34,6% in der Region Süd und 8,4% in der Region Ost tätig. In der Gruppe der psychologischen Psychotherapeuten waren es 38,0% in der KV-Region West, 11,6% in der Region Nord, 26,3% in der Region Süd und 10,6% in der Region Ost 18 . Somit ist davon auszugehen, dass die Befragungsergebnisse auf die ambulante Versorgung übertragbar sind. Die Angaben in der Befragung basieren auf Selbstauskünften und die Ergebnisse stellen somit eine subjektive Einschätzung der befragten Psychotherapeuten dar. Die Länge des Fragebogens könnte sich negativ auf die Teilnahmebereitschaft ausgewirkt haben. Aufgrund des geringen Anteils an vermittelten Terminen im Bereich der kinder- und jugendpsychotherapeutischen Leistungen, wurden die Befragungsergebnisse von diesem Kollektiv in der vorliegenden Publikation nicht darstellt.

## Konsequenzen für Klinik und Praxis

- 25,0% der Psychotherapeuten stimmten der Aussage zu, dass die TSS als zentrale Anlaufstelle gut funktioniere, woraus sich Optimierungspotenzial ableiten lässt.
- Psychotherapeuten, die bereits mit der TSS zusammengearbeitet haben, waren mehrheitlich zufrieden, jedoch berichteten 65,5% der Befragten davon, keine Kapazitäten zu haben, um Termine an die TSS zu melden.
- Lösungsansätze könnten in Maßnahmen zur Steigerung der Terminverfügbarkeit oder der Terminverbindlichkeit liegen.

## Fördermittel

Innovationsausschuss beim Gemeinsamen Bundesausschuss — 01VSF19006

## Footnotes

^1^ Zusätzlich wurden rund 10% der Termine im Kinder- und Jugendbereich vermittelt.

## Notes

Zusatzmaterial zum Artikel Additional Information (Literature)
