# Supplementary material for: TSS in the care of people with mental illness – results of a survey of psychotherapists
Source: Psychiatr Prax. 2026 Apr 22;53(3):133–9. [Article in German] doi: 10.1055/a-2797-1205 (PMC13288593; doi:10.1055/a-2797-1205)
Supplement: Supplementary file 1 — Zusatzmaterial zum Artikel [file 10-1055-a-2797-1205_lit.pdf]

Anhang 1: Erfahrungen mit und Einschätzung zur TSS

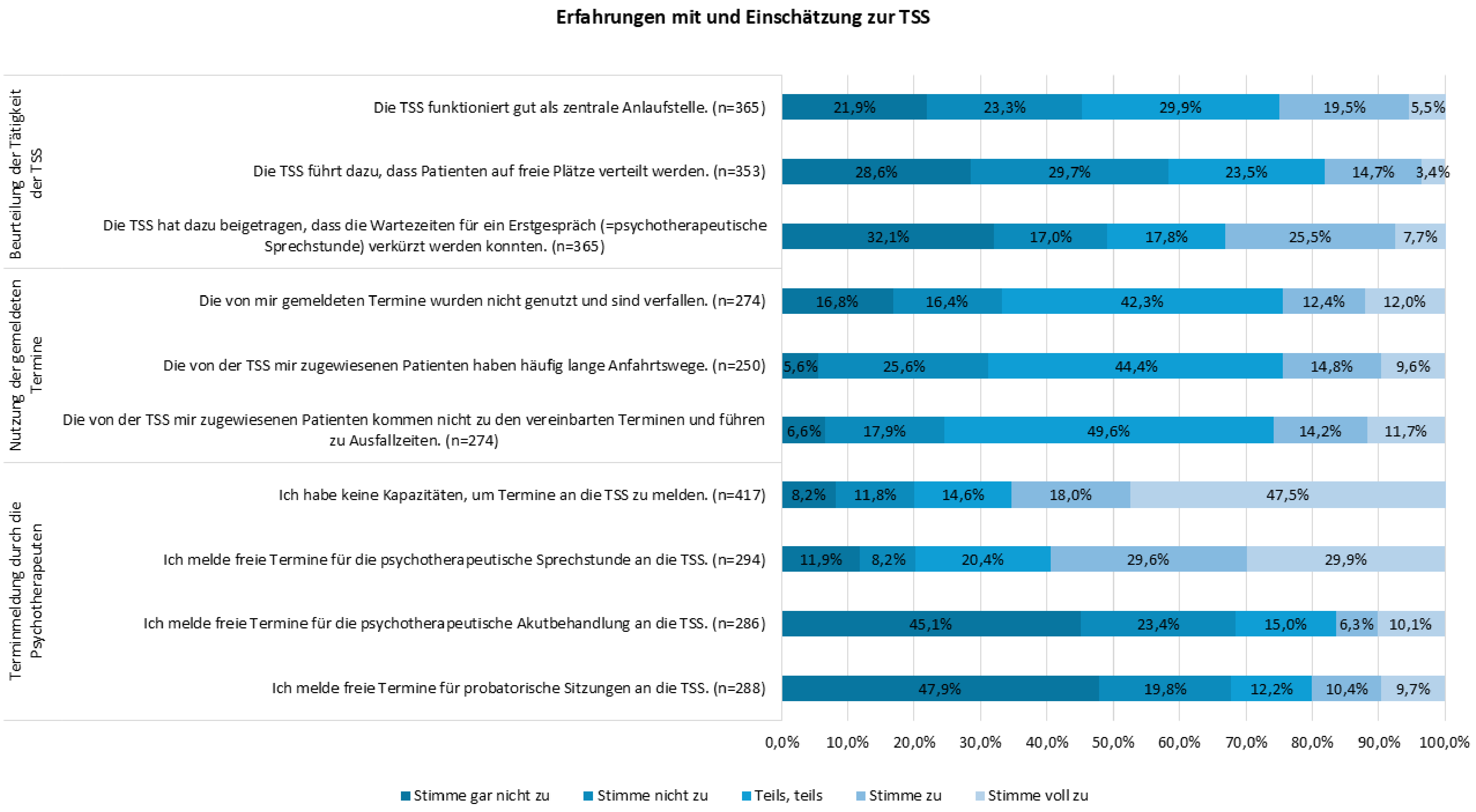

Psychiat Prax | DOI 10.1055/a-2797-1205 | © 2026. The Author(s).

Anhang 2: Beurteilung der Zufriedenheit mit der TSS

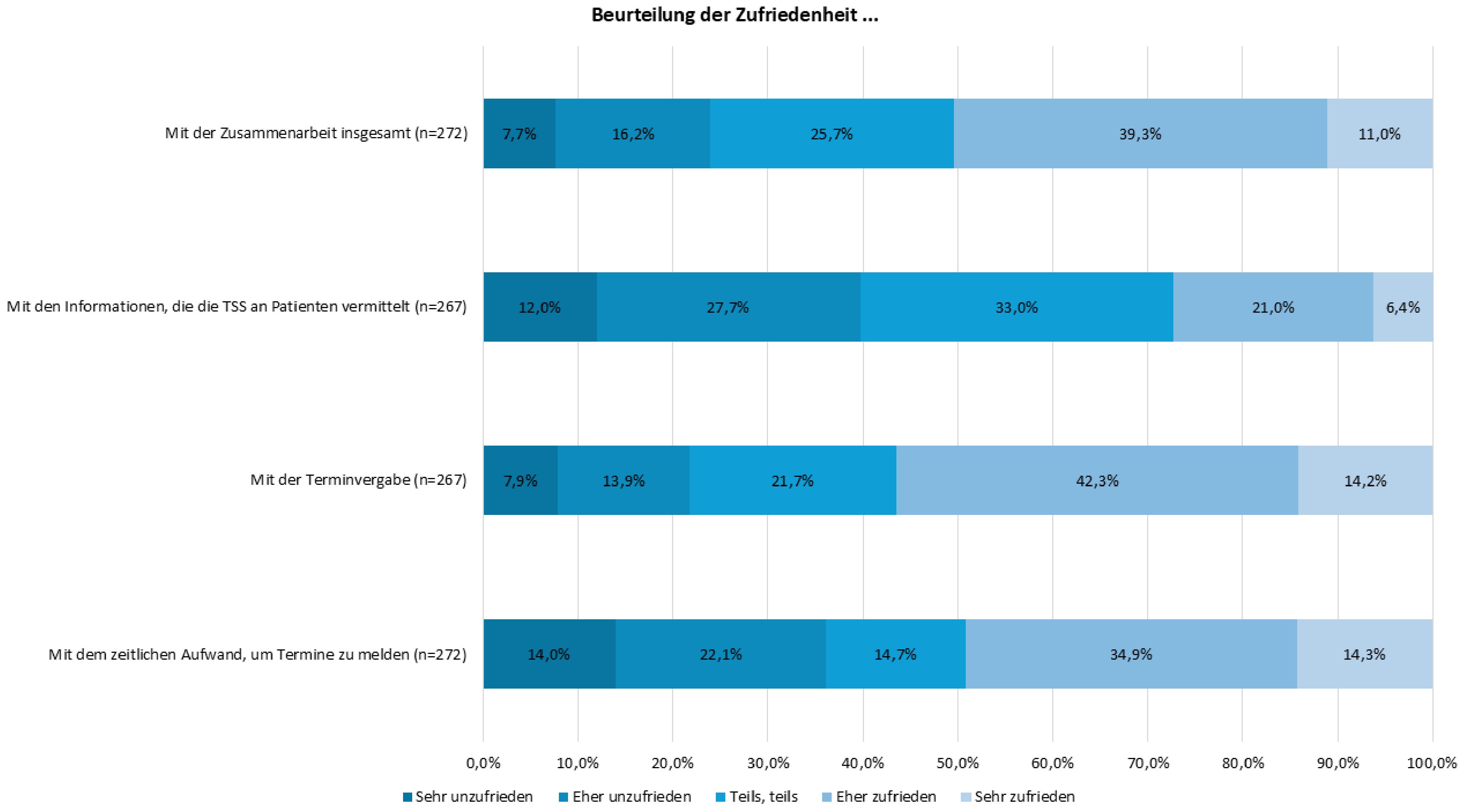

Anhang 3: Subgruppenanalysen zu den Erfahrungen mit und Einschätzung zur TSS stratifiziert nach den Merkmalen der Psychotherapeuten(1)

			**Die TSS funktioniert gut als zentrale Anlaufstelle.**						**Die TSS führt dazu, dass Patienten auf freie Plätze verteilt werden.**						**Die TSS hat dazu beigetragen, dass die Wartezeiten für ein Erstgespräch (=ptS) verkürzt werden konnten.**				
			**Stimme gar nicht zu N (%)**	**Stimme nicht zu N (%)**	**Teils, teils N (%)**	**Stimme zu N (%)**	**Stimme (voll) zu N (%)**		**Stimme gar nicht zu N (%)**	**Stimme nicht zu N (%)**	**Teils, teils N (%)**	**Stimme zu N (%)**	**Stimme (voll) zu N (%)**		**Stimme gar nicht zu N (%)**	**Stimme nicht zu N (%)**	**Teils, teils N (%)**	**Stimme zu N (%)**	**Stimme (voll) zu N (%)**
**Alter[1]**			**(N=79)**	**(N=85)**	**(N=106)**	**(N=71)**	**(N=20)**		**(N=101)**	**(N=102)**	**(N=82)**	**(N=52)**	**(N=12)**		**(N= 115)**	**(N=61)**	**(N=64)**	**(N=93)**	**(N=28)**
	21 - 40		10 (12,7)	8 (9,4)	11 (10,4)	5 (7,0)	4 (20,0)		10 (9,9)	12 (11,8)	8 (9,8)	9 (17,3)	1 (8,3)		12 (10,4)	2 (3,3)	7 (10,9)	14 (15,1)	2 (7,1)
	41 - 50		20 (25,3)	17 (20,0)	27 (25,5)	19 (26,8)	1 (5,0)		24 (23,8)	29 (28,4)	20 (24,4)	7 (13,5)	3 (25,0)		29 (25,2)	19 (31,1)	17 (26,6)	18 (19,4)	5 (17,9)
	51 - 60		24 (30,4)	34 (40,0)	33 (31,1)	26 (36,6)	7 (35,0)		34 (33,7)	32 (31,4)	22 (26,8)	20 (38,5)	5 (41,7)		39 (33,9)	19 (31,1)	16 (25,0)	34 (36,6)	11 (39,3)
	über 60		25 (31,6)	26 (30,6)	35 (33,0)	21 (29,6)	8 (40,0)		33 (32,7)	29 (28,4)	32 (39,0)	16 (30,8)	3 (25,0)		35 (30,4)	21 (34,4)	24 (37,5)	27 (29,0)	10 (35,7)
	Fehlend	*86*						*98*						*86*					
			p n.s.; Cramer's V: ,092						p n.s.; Cramer's V: ,093						p n.s.; Cramer's V: ,102				
**Geschlecht**			**(N=77)**	**(N=82)**	**(N=102)**	**(N=68)**	**(N=20)**		**(N=98)**	**(N=100)**	**(N=77)**	**(N=52)**	**(N=12)**		**(N=109)**	**(N=60)**	**(N=65)**	**(N=88)**	**(N=27)**
	Männlich		22 (28,6)	15 (18,3)	25 (24,5)	15 (22,1)	7 (35,0)		26 (26,5)	18 (18,0)	17 (22,1)	16 (30,8)	6 (50,0)		28 (25,7)	10 (16,7)	20 (30,8)	17 (19,3)	6 (22,2)
	Weiblich		55 (71,4)	67 (81,7)	77 (75,5)	53 (77,9)	13 (65,0)		72 (73,5)	82 (82,0)	60 (77,9)	36 (69,2)	6 (50,0)		81 (74,3)	50 (83,3)	45 (69,2)	71 (80,7)	21 (77,8)
	Fehlend	*98*						*108*						*98*					
			p n.s.; Phi: ,105						p n.s.; Phi: ,154						p n.s.; Phi: ,116				
**Studiengang**			**(N=77)**	**(N=85)**	**(N=103)**	**(N=71)**	**(N=20)**		**(N=101)**	**(N=102)**	**(N=79)**	**(N=51)**	**(N=12)**		**(N=114)**	**(N=61)**	**(N=62)**	**(N=91)**	**(N=28)**
	Medizin		6 (7,8)	9 (10,6)	9 (8,7)	10 (14,1)	2 (10,0)		9 (8,9)	8 (7,8)	6 (7,6)	9 (17,6)	1 (8,3)		8 (7,0)	6 (9,8)	2 (3,2)	15 (16,5)	3 (10,7)
	Psychologie		69 (89,6)	74 (87,1)	93 (90,3)	61 (85,9)	18 (90,0)		89 (88,1)	93 (91,2)	73 (92,4)	42 (82,4)	11 (91,7)		104 (91,2)	53 (86,9)	60 (96,8)	76 (83,5)	25 (89,3)
	Anderes Studium		2 (2,6)	2 (2,4)	1 (1,0)	0 (0,0)	0 (0,0)		3 (3,0)	1 (1,0)	0 (0,0)	0 (0,0)	0 (0,0)		2 (1,8)	2 (3,3)	0 (0,0)	0 (0,0)	0 (0,0)
	Fehlend	*91*						*102*						*91*					
			p n.s.; Cramer's V: ,080						p n.s.; Cramer's V: ,115						p n.s.; Cramer's V: ,139				
**PT-Verfahren**			**(N=78)**	**(N=85)**	**(N=107)**	**(N=71)**	**(N=20)**		**(N=101)**	**(N=102)**	**(N=83)**	**(N=52)**	**(N=12)**		**(N=115)**	**(N=62)**	**(N=65)**	**(N=91)**	**(N=28)**
	Nur Tiefenpsychologisch		16 (20,5)	15 (17,6)	30 (28,0)	24 (33,8)	4 (20,0)		22 (21,8)	23 (22,5)	22 (26,5)	14 (26,9)	2 (16,7)		22 (19,1)	16 (25,8)	15 (23,1)	28 (30,8)	9 (32,1)
	Nur Verhaltenstherapie		48 (61,5)	54 (63,5)	58 (54,2)	31 (43,7)	12 (60,0)		56 (55,4)	64 (62,7)	40 (48,2)	31 (59,6)	9 (75,0)		73 (63,5)	30 (48,4)	39 (60,0)	48 (52,7)	11 (39,3)
	Nur Analytisch und Tiefenpsychologisch		9 (11,5)	11 (12,9)	13 (12,1)	13 (18,3)	3 (15,0)		14 (13,9)	11 (10,8)	17 (20,5)	5 (9,6)	0 (0,0)		12 (10,4)	14 (22,6)	8 (12,3)	11 (12,1)	5 (17,9)
	Fehlend	*86*						*97*						*86*					
			p n.s.; Cramer's V: ,099						p n.s.; Cramer's V: ,108						p n.s.; Cramer's V: ,117				
**KV-Region[2]**			**(N=80)**	**(N=84)**	**(N=109)**	**(N=70)**	**(N=20)**		**(N=101)**	**(N=104)**	**(N=83)**	**(N=52)**	**(N=12)**		**(N=116)**	**(N=61)**	**(N=65)**	**(N=93)**	**(N=28)**
	Region West		28 (35,0)	34 (40,5)	37 (33,9)	20 (28,6)	6 (30,0)		29 (28,7)	40 (38,5)	26 (31,3)	20 (38,5)	6 (50,0)		37 (31,9)	20 (32,8)	22 (33,8)	36 (38,7)	9 (32,1)
	Region Nord		18 (22,5)	8 (9,5)	8 (7,3)	9 (12,9)	1 (5,0)		17 (16,8)	13 (12,5)	7 (8,4)	6 (11,5)	0 (0,0)		23 (19,8)	5 (8,2)	3 (4,6)	10 (10,8)	4 (14,3)
	Region Süd		27 (33,8)	32 (38,1)	25 (22,9)	19 (27,1)	5 (25,0)		31 (30,7)	30 (28,8)	25 (30,1)	13 (25,0)	1 (8,3)		40 (34,5)	22 (36,1)	23 (35,4)	20 (21,5)	1 (3,6)
	Region Ost		3 (3,8)	3 (3,6)	15 (13,8)	10 (14,3)	1 (5,0)		7 (6,9)	11 (10,6)	9 (10,8)	9 (17,3)	1 (8,3)		5 (4,3)	8 (13,1)	6 (9,2)	12 (12,9)	4 (14,3)
	Berlin		1 (1,3)	6 (7,1)	11 (10,1)	8 (11,4)	6 (30,0)		7 (6,9)	5 (4,8)	10 (12,0)	3 (5,8)	4 (33,3)		8 (6,9)	3 (4,9)	6 (9,2)	9 (9,7)	4 (14,3)
	Hamburg		3 (3,8)	1 (1,2)	13 (11,9)	4 (5,7)	1 (5,0)		10 (9,9)	5 (4,8)	6 (7,2)	1 (1,9)	0 (0,0)		3 (2,6)	3 (4,9)	5 (7,7)	6 (6,5)	6 (21,4)
	Fehlend	*84*						*95*						*84*					
			p <0,001; Cramer's V: ,193						p n.s.; Cramer's V: ,147						p <0,01; Cramer's V: ,172				
**Gemeindegröße**			**(N=80)**	**(N=85)**	**(N=106)**	**(N=70)**	**(N=20)**		**(N=101)**	**(N=103)**	**(N=81)**	**(N=52)**	**(N=12)**		**(N=116)**	**(N=61)**	**(N=64)**	**(N=92)**	**(N=28)**
	Landgemeinde		4 (5,0)	13 (15,3)	4 (3,8)	6 (8,6)	1 (5,0)		5 (5,0)	13 (12,6)	8 (9,9)	4 (7,7)	0 (0,0)		8 (6,9)	9 (14,8)	6 (9,4)	8 (8,7)	0 (0,0)
	Kleinstadt		18 (22,5)	12 (14,1)	12 (11,3)	12 (17,1)	2 (10,0)		16 (15,8)	16 (15,5)	8 (9,9)	13 (25,0)	0 (0,0)		23 (19,8)	7 (11,5)	7 (10,9)	19 (20,7)	3 (10,7)
	Mittelstadt		25 (31,3)	25 (29,4)	21 (19,8)	11 (15,7)	7 (35,0)		27 (26,7)	26 (25,2)	16 (19,8)	11 (21,2)	5 (41,7)		37 (31,9)	16 (26,2)	8 (12,5)	18 (19,6)	4 (14,3)
	Großstadt		33 (41,3)	35 (41,2)	69 (65,1)	41 (58,6)	10 (50,0)		53 (52,5)	48 (46,6)	49 (60,5)	24 (46,2)	7 (58,3)		48 (41,4)	29 (47,5)	43 (67,2)	47 (51,1)	21 (75,0)
	Fehlend	*86*						*98*						*86*					
			p <0,01; Cramer's V: ,160						p n.s.; Cramer's V: ,124						p <0,01; Cramer's V: ,158				
**Patientengruppe[3]**			**(N=78)**	**(N=85)**	**(N=107)**	**(N=71)**	**(N=20)**		**(N=101)**	**(N=102)**	**(N=83)**	**(N=52)**	**(N=12)**		**(N=115)**	**(N=62)**	**(N=65)**	**(N=91)**	**(N=28)**
	Ausschließlich Erwachsene		70 (89,7)	74 (87,1)	93 (86,9)	67 (94,4)	18 (90,0)		91 (90,1)	92 (90,2)	73 (88,0)	48 (92,3)	10 (83,3)		100 (87,0)	58 (93,5)	59 (90,8)	83 (91,2)	25 (89,3)
	Erwachsene/Kinder/Jugendliche		8 (10,3)	11 (12,9)	14 (13,1)	4 (5,6)	2 (10,0)		10 (9,9)	10 (9,8)	10 (12,0)	4 (7,7)	2 (16,7)		15 (13,0)	4 (6,5)	6 (9,2)	8 (8,8)	3 (10,7)
	Fehlend	*86*						*97*						*86*					
			p n.s.; Phi: ,091						p n.s.; Phi: ,059						p n.s.; Phi: ,079				

[1]Aufgrund geringer Fallzahlen wurden die Altersgruppen 21-30 und 31-40 zusammengefasst.

[2]KV-Region West (Westfalen-Lippe, Nordrhein, Hessen, Rheinland-Pfalz, Saarland), KV-Region Nord (Schleswig-Holstein, Niedersachsen, Bremen), KV-Region Süd (Baden-Württemberg, Bayern), KV-Region Ost (Mecklenburg-Vorpommern, Brandenburg, Sachsen, Sachsen-Anhalt, Thüringen).
[3]Wenn beide Gruppen mindestens einen Anteil von ca. einem Drittel der betreuten Patienten ausmacht.

Anhang 4: Subgruppenanalysen zu den Erfahrungen mit und Einschätzung zur TSS stratifiziert nach den Merkmalen der Psychotherapeuten (2)

			**Die von mir gemeldeten Termine wurden nicht genutzt und sind verfallen.**						**Die von der TSS mir zugewiesenen Patienten haben häufig lange Anfahrtswege.**						**Die von der TSS mir zugewiesenen Patienten kommen nicht zu den vereinbarten Terminen und führen zu Ausfallzeiten.**				
			**Stimme gar nicht zu N (%)**	**Stimme nicht zu N (%)**	**Teils, teils N (%)**	**Stimme zu N (%)**	**Stimme (voll) zu N (%)**		**Stimme gar nicht zu N (%)**	**Stimme nicht zu N (%)**	**Teils, teils N (%)**	**Stimme zu N (%)**	**Stimme (voll) zu N (%)**		**Stimme gar nicht zu N (%)**	**Stimme nicht zu N (%)**	**Teils, teils N (%)**	**Stimme zu N (%)**	**Stimme (voll) zu N (%)**
**Alter[1]**			**(N=46)**	**(N=44)**	**(N=114)**	**(N=34)**	**(N=32)**		**(N=14)**	**(N=62)**	**(N=111)**	**(N=37)**	**(N=23)**		**(N=18)**	**(N=48)**	**(N=135)**	**(N=39)**	**(N=31)**
	21 - 40		6 (13,0)	6 (13,6)	18 (15,8)	0 (0,0)	2 (6,3)		2 (14,3)	6 (9,7)	13 (11,7)	6 (16,2)	5 (21,7)		2 (11,1)	4 (8,3)	17 (12,6)	6 (15,4)	3 (9,7)
	41 - 50		9 (19,6)	12 (27,3)	21 (18,4)	12 (35,3)	10 (31,3)		0 (0,0)	16 (25,8)	29 (26,1)	8 (21,6)	8 (34,8)		3 (16,7)	11 (22,9)	28 (20,7)	13 (33,3)	9 (29,0)
	51 - 60		17 (37,0)	12 (27,3)	47 (41,2)	13 (38,2)	12 (37,5)		5 (35,7)	21 (33,9)	44 (39,6)	10 (27,0)	4 (17,4)		7 (38,9)	19 (39,6)	47 (34,8)	12 (30,8)	11 (35,5)
	über 60		14 (30,4)	14 (31,8)	28 (24,6)	9 (26,5)	8 (25,0)		7 (50,0)	19 (30,6)	25 (22,5)	13 (35,1)	6 (26,1)		6 (33,3)	14 (29,2)	43 (31,9)	8 (20,5)	8 (25,8)
	Fehlend	*177*						*200*						*176*					
			p n.s.; Cramer's V: ,130						p n.s.; Cramer's V: ,142						p n.s.; Cramer's V: ,086				
**Geschlecht**			**(N=42)**	**(N=44)**	**(N=109)**	**(N=33)**	**(N=31)**		**(N=12)**	**(N=59)**	**(N=108)**	**(N=36)**	**(N=22)**		**(N=17)**	**(N=47)**	**(N=128)**	**(N=38)**	**(N=30)**
	Männlich		12 (28,6)	5 (11,4)	25 (22,9)	8 (24,2)	9 (29,0)		5 (41,7)	12 (20,3)	24 (22,2)	11 (30,6)	4 (18,2)		5 (29,4)	5 (10,6)	34 (26,6)	9 (23,7)	7 (23,3)
	Weiblich		30 (71,4)	39 (88,6)	84 (77,1)	25 (75,8)	22 (71,0)		7 (58,3)	47 (79,7)	84 (77,8)	25 (69,4)	18 (81,8)		12 (70,6)	42 (89,4)	94 (73,4)	29 (76,3)	23 (76,7)
	Fehlend	*188*						*210*						*187*					
			p n.s.; Phi: ,136						p n.s.; Phi: ,129						p n.s.; Phi: ,144				
**Studiengang**			**(N=42)**	**(N=44)**	**(N=115)**	**(N=33)**	**(N=32)**		**(N=14)**	**(N=63)**	**(N=110)**	**(N=36)**	**(N=23)**		**(N=17)**	**(N=48)**	**(N=134)**	**(N=38)**	**(N=32)**
	Medizin		3 (7,1)	4 (9,1)	12 (10,4)	3 (9,1)	6 (18,8)		2 (14,3)	4 (6,3)	14 (12,7)	6 (16,7)	1 (4,3)		0 (0,0)	4 (8,3)	18 (13,4)	1 (2,6)	5 (15,6)
	Psychologie		39 (92,9)	39 (88,6)	101 (87,8)	28 (84,8)	26 (81,3)		12 (85,7)	58 (92,1)	93 (84,5)	30 (83,3)	21 (91,3)		16 (94,1)	42 (87,5)	114 (85,1)	36 (94,7)	27 (84,4)
	Anderes Studium		0 (0,0)	1 (2,3)	2 (1,7)	2 (6,1)	0 (0,0)		0 (0,0)	1 (1,6)	3 (2,7)	0 (0,0)	1 (4,3)		1 (5,9)	2 (4,2)	2 (1,5)	1 (2,6)	0 (0,0)
	Fehlend	*181*						*201*						*178*					
			p n.s.; Cramer's V: ,119						p n.s.; Cramer's V: ,110						p n.s.; Cramer's V: ,133				
**PT-Verfahren**			**(N=44)**	**(N=44)**	**(N=116)**	**(N=33)**	**(N=33)**		**(N=14)**	**(N=63)**	**(N=111)**	**(N=37)**	**(N=24)**		**(N=18)**	**(N=48)**	**(N=135)**	**(N=39)**	**(N=32)**
	Nur Tiefenpsychologisch		12 (27,3)	16 (36,4)	25 (21,6)	9 (27,3)	6 (18,2)		5 (35,7)	21 (33,3)	30 (27,0)	6 (16,2)	4 (16,7)		4 (22,2)	18 (37,5)	34 (25,2)	10 (25,6)	6 (18,8)
	Nur Verhaltenstherapie		26 (59,1)	18 (40,9)	71 (61,2)	18 (54,5)	20 (60,6)		6 (42,9)	28 (44,4)	63 (56,8)	27 (73,0)	14 (58,3)		9 (50,0)	25 (52,1)	73 (54,1)	20 (51,3)	21 (65,6)
	Nur Analytisch und Tiefenpsychologisch		6 (13,6)	9 (20,5)	12 (10,3)	3 (9,1)	4 (12,1)		1 (7,1)	13 (20,6)	12 (10,8)	2 (5,4)	4 (16,7)		4 (22,2)	5 (10,4)	20 (14,8)	6 (15,4)	2 (6,3)
	Fehlend	*177*						*198*						*175*					
			p n.s.; Cramer's V: ,133						p n.s.; Cramer's V: ,154						p n.s.; Cramer's V: ,115				
**KV-Region[2]**			**(N=46)**	**(N=44)**	**(N=116)**	**(N=34)**	**(N=33)**		**(N=14)**	**(N=64)**	**(N=109)**	**(N=37)**	**(N=24)**		**(N=18)**	**(N=49)**	**(N=134)**	**(N=39)**	**(N=32)**
	Region West		13 (28,3)	14 (31,8)	46 (39,7)	13 (38,2)	7 (21,2)		2 (14,3)	24 (37,5)	35 (32,1)	17 (45,9)	8 (33,3)		3 (16,7)	17 (34,7)	47 (35,1)	15 (38,5)	11 (34,4)
	Region Nord		11 (23,9)	8 (18,2)	7 (6,0)	0 (0,0)	3 (9,1)		1 (7,1)	9 (14,1)	9 (8,3)	1 (2,7)	6 (25,0)		5 (27,8)	4 (8,2)	13 (9,7)	3 (7,7)	4 (12,5)
	Region Süd		6 (13,0)	7 (15,9)	31 (26,7)	13 (38,2)	19 (57,6)		5 (35,7)	8 (12,5)	30 (27,5)	11 (29,7)	9 (37,5)		6 (33,3)	11 (22,4)	31 (23,1)	15 (38,5)	9 (28,1)
	Region Ost		9 (19,6)	1 (2,3)	13 (11,2)	4 (11,8)	1 (3,0)		1 (7,1)	6 (9,4)	19 (17,4)	2 (5,4)	0 (0,0)		0 (0,0)	4 (8,2)	19 (14,2)	4 (10,3)	3 (9,4)
	Berlin		4 (8,7)	8 (18,2)	12 (10,3)	1 (2,9)	2 (6,1)		2 (14,3)	6 (9,4)	12 (11,0)	4 (10,8)	1 (4,2)		3 (16,7)	7 (14,3)	13 (9,7)	1 (2,6)	3 (9,4)
	Hamburg		3 (6,5)	6 (13,6)	7 (6,0)	3 (8,8)	1 (3,0)		3 (21,4)	11 (17,2)	4 (3,7)	2 (5,4)	0 (0,0)		1 (5.6)	6 (12,2)	11 (8,2)	1 (2,6)	2 (6,3)
	Fehlend	*174*						*199*						*175*					
			p <0,001; Cramer's V: ,223						p <0,01; Cramer's V: ,206						p n.s.; Cramer's V: ,140				
**Gemeindegröße**			**(N=46)**	**(N=45)**	**(N=116)**	**(N=34)**	**(N=33)**		**(N=14)**	**(N=64)**	**(N=110)**	**(N=37)**	**(N=24)**		**(N=18)**	**(N=49)**	**(N=135)**	**(N=39)**	**(N=32)**
	Landgemeinde		1 (2,2)	4 (8,9)	8 (6,9)	2 (5,9)	1 (3,0)		0 (0,0)	5 (7,8)	8 (7,3)	2 (5,4)	2 (8,3)		3 (16,7)	4 (8,2)	7 (5,2)	3 (7,7)	0 (0,0)
	Kleinstadt		5 (10,9)	2 (4,4)	17 (14,7)	6 (17,6)	5 (15,2)		1 (7,1)	5 (7,8)	14 (12,7)	5 (13,5)	7 (29,2)		0 (0,0)	4 (8,2)	19 (14,1)	7 (17,9)	7 (21,9)
	Mittelstadt		14 (30,4)	6 (13,3)	25 (21,6)	9 (26,5)	12 (36,4)		6 (42,9)	8 (12,5)	29 (26,4)	10 (27,0)	5 (20,8)		5 (27,8)	9 (18,4)	30 (22,2)	10 (25,6)	8 (25,0)
	Großstadt		26 (56,5)	33 (73,3)	66 (56,9)	17 (50,0)	15 (45,5)		7 (50,0)	46 (71,9)	59 (53,6)	20 (54,1)	10 (41,7)		10 (55,6)	32 (65,3)	79 (58,5)	19 (48,7)	17 (53,1)
	Fehlend	*173*						*198*						*174*					
			p n.s.; Cramer's V: ,134						p n.s.; Cramer's V: ,155						p n.s.; Cramer's V: ,129				
**Patientengruppe[3]**			**(N=44)**	**(N=44)**	**(N=116)**	**(N=33)**	**(N=33)**		**(N=14)**	**(N=63)**	**(N=111)**	**(N=37)**	**(N=24)**		**(N=18)**	**(N=48)**	**(n=135)**	**(N=39)**	**(N=32)**

	Ausschließlich Erwachsene		40 (90,9)	42 (95,5)	105 (90,5)	28 (84,8)	28 (84,8)		13 (92,9)	57 (90,5)	101 (91,0)	33 (89,2)	22 (91,7)		17 (94,4)	45 (93,8)	121 (89,6)	34 (87,2)	28 (87,5)
	Erwachsene/Kinder/Jugendliche		4 (9,1)	2 (4,5)	11 (9,5)	5 (15,2)	5 (15,2)		1 (7,11)	6 (9,5)	10 (9,0)	4 (10,8)	2 (8,3)		1 (5,6)	3 (6,3)	14 (10,4)	5 (12,8)	4 (12,5)
	Fehlend	*177*						*198*						*175*					
			p n.s.; Phi: ,113						p n.s.; Phi: ,030						p n.s.; Phi: ,080				

[1]Aufgrund geringer Fallzahlen wurden die Altersgruppen 21-30 und 31-40 zusammengefasst.
[2]KV-Region West (Westfalen-Lippe, Nordrhein, Hessen, Rheinland-Pfalz, Saarland), KV-Region Nord (Schleswig-Holstein, Niedersachsen, Bremen), KV-Region Süd (Baden-Württemberg, Bayern), KV-Region Ost (Mecklenburg-Vorpommern, Brandenburg, Sachsen, Sachsen-Anhalt, Thüringen).
[3]Wenn beide Gruppen mindestens einen Anteil von ca. einem Drittel der betreuten Patienten ausmacht.

Anhang 5: Subgruppenanalysen zu den Erfahrungen mit und Einschätzung zur TSS stratifiziert nach den Merkmalen der Psychotherapeuten (3)

			**Ich habe keine Kapazitäten, um Termine an die TSS zu melden.**						**Ich melde freie Termine für die psychotherapeutische Sprechstunde an die TSS.**						**Ich melde freie Termine für die psychotherapeutische Akutbehandlung an die TSS.**						**Ich melde freie Termine für probatorische Sitzungen an die TSS.**				
			**Stimme gar nicht zu N (%)**	**Stimme nicht zu N (%)**	**Teils, teils N (%)**	**Stimme zu N (%)**	**Stimme (voll) zu N (%)**		**Stimme gar nicht zu N (%)**	**Stimme nicht zu N (%)**	**Teils, teils N (%)**	**Stimme zu N (%)**	**Stimme (voll) zu N (%)**		**Stimme gar nicht zu N (%)**	**Stimme nicht zu N (%)**	**Teils, teils N (%)**	**Stimme zu N (%)**	**Stimme (voll) zu N (%)**		**Stimme gar nicht zu N (%)**	**Stimme nicht zu N (%)**	**Teils, teils N (%)**	**Stimme zu N (%)**	**Stimme (voll) zu N (%)**
**Alter[1]**			**(N=33)**	**(N=48)**	**(N=61)**	**(N=73)**	**(N=198)**		**(N=34)**	**(N=24)**	**(N=59)**	**(N=87)**	**(N=86)**		**(N=127)**	**(N=67)**	**(N=41)**	**(N=18)**	**(N=29)**		**(N=136)**	**(N=57)**	**(N=34)**	**(N=29)**	**(N=28)**
	21 - 40		5 (15,2)	7 (14,6)	5 (8,2)	12 (16,4)	13 (6,6)		4 (11,8)	3 (12,5)	7 (11,9)	11 (12,6)	9 (10,5)		16 (12,6)	8 (11,9)	8 (19,5)	2 (11,1)	0 (0,0)		18 (13,2)	6 (10,5)	7 (20,6)	2 (6,9)	1 (3,6)
	41 - 50		9 (27,3)	13 (27,1)	11 (18,0)	15 (20,5)	47 (23,7)		6 (17,6)	6 (25,0)	12 (20,3)	20 (23,0)	25 (29,1)		35 (27,6)	17 (25,4)	5 (12,2)	3 (16,7)	10 (34,5)		36 (26,5)	13 (22,8)	4 (11,8)	6 (20,7)	10 (35,7)
	51 - 60		9 (27,3)	12 (25,0)	26 (42,6)	23 (31,5)	72 (36,4)		10 (29,4)	8 (33,3)	23 (39,0)	30 (34,5)	33 (38,4)		40 (31,5)	22 (32,8)	19 (46,3)	7 (38,9)	12 (41,4)		47 (34,6)	19 (33,3)	13 (38,2)	9 (31,0)	11 (39,3)
	über 60		10 (30,3)	16 (33,3)	19 (31,1)	23 (31,5)	66 (33,3)		14 (41,2)	7 (29,2)	17 (28,8)	26 (29,9)	19 (22,1)		36 (28,3)	20 (29,9)	9 (22,0)	6 (33,3)	7 (24,1)		35 (25,7)	19 (33,3)	10 (29,4)	12 (41,4)	6 (21,4)
	Fehlend	*34*						*157*						*165*						*163*					
			p n.s.; Cramer's V: ,100						p n.s.; Cramer's V: ,084						p n.s.; Cramer's V: ,126						p n.s.; Cramer's V: ,120				
**Geschlecht**			**(N=32)**	**(N=46)**	**(N=58)**	**(N=70)**	**(N=195)**		**(N=31)**	**(N=24)**	**(N=57)**	**(N=83)**	**(N=83)**		**(N=122)**	**(N=66)**	**(N=39)**	**(N=16)**	**(N=28)**		**(N=130)**	**(N=55)**	**(N=35)**	**(N=28)**	**(N=25)**
	Männlich		9 (28,1)	10 (21,7)	12 (20,7)	12 (17,1)	51 (26,2)		14 (45,2)	6 (25,0)	15 (26,3)	11 (13,3)	21 (25,3)		32 (26,2)	13 (19,7)	7 (17,9)	5 (31,3)	8 (28,6)		34 (26,2)	11 (20,0)	11 (31,4)	4 (14,3)	7 (28,0)
	Weiblich		23 (71,9)	36 (78,3)	46 (79,3)	58 (82,9)	144 (73,8)		17 (54,8)	18 (75,0)	42 (73,7)	72 (86,7)	62 (74,7)		90 (73,8)	53 (80,3)	32 (82,1)	11 (68,8)	20 (71,4)		96 (73,8)	44 (80,0)	24 (68,6)	24 (85,7)	18 (72,0)
	Fehlend	*46*						*169*						*176*						*174*					
			p n.s.; Phi: ,087						p <0,05; Phi: ,217						p n.s.; Phi: ,097						p n.s.; Phi: ,112				
**Studiengang**			**(N=33)**	**(N=48)**	**(N=59)**	**(N=72)**	**(N=194)**		**(N=33)**	**(N=23)**	**(N=56)**	**(N=86)**	**(N=88)**		**(N=127)**	**(N=64)**	**(N=42)**	**(N=16)**	**(N=29)**		**(N=135)**	**(N=54)**	**(N=33)**	**(N=30)**	**(N=28)**
	Medizin		3 (9,1)	5 (10,4)	9 (15,3)	12 (16,7)	9 (4,6)		4 (12,1)	0 (0,0)	10 (17,9)	9 (10,5)	8 (9,1)		13 (10,2)	6 (9,4)	7 (16,7)	0 (0,0)	2 (6,9)		14 (10,4)	7 (13,0)	5 (15,2)	2 (6,7)	0 (0,0)
	Psychologie		29 (87,9)	41 (85,4)	49 (83,1)	60 (83,3)	183 (94,3)		29 (87,9)	23 (100,0)	45 (80,4)	74 (86,0)	78 (88,6)		112 (88,2)	58 (90,6)	34 (81,0)	14 (87,5)	26 (89,7)		119 (88,1)	46 (85,2)	27 (81,8)	27 (90,0)	27 (96,4)
	Anderes Studium		1 (3,0)	2 (4,2)	1 (1,7)	0 (0,0)	2 (1,0)		0 (0,0)	0 (0,0)	1 (1,8)	3 (3,5)	2 (2,3)		2 (1,6)	0 (0,0)	1 (2,4)	2 (12,5)	1 (3,4)		2 (1,5)	1 (1,9)	1 (3,0)	1 (3,3)	1 (3,6)
	Fehlend	*41*						*161*						*169*						*167*					
			p <0.05; Cramer's V: ,142						p n.s.; Cramer's V: ,119						p n.s.; Cramer's V: ,157						p n.s.; Cramer's V: ,102				
**PT-Verfahren**			**(N=33)**	**(N=48)**	**(N=60)**	**(N=74)**	**(N=197)**		**(N=33)**	**(N=24)**	**(N=59)**	**(N=86)**	**(N=88)**		**(N=127)**	**(N=66)**	**(N=43)**	**(N=17)**	**(N=29)**		**(N=136)**	**(N=56)**	**(N=34)**	**(N=30)**	**(N=28)**
	Nur Tiefenpsychologisch		8 (24,2)	10 (20,8)	21 (35,0)	22 (29,7)	45 (22,8)		8 (24,2)	3 (12,5)	19 (32,2)	25 (29,1)	18 (20,5)		31 (24,4)	16 (24,2)	13 (30,2)	3 (17,6)	6 (20,7)		34 (25,0)	16 (28,6)	8 (23,5)	9 (30,0)	5 (17,9)
	Nur Verhaltenstherapie		19 (57,6)	28 (58,3)	30 (50,0)	41 (55,4)	114 (57,9)		16 (48,5)	14 (58,3)	29 (49,2)	48 (55,8)	55 (62,5)		72 (56,7)	37 (56,1)	23 (53,5)	11 (64,7)	18 (62,1)		78 (57,4)	29 (51,8)	21 (61,8)	14 (46,7)	20 (71,4)
	Nur Analytisch und Tiefenpsychologisch		3 (9,1)	7 (14,6)	7 (11,7)	7 (9,5)	29 (14,7)		6 (18,2)	5 (20,8)	8 (13,6)	10 (11,6)	10 (11,4)		20 (15,7)	7 (10,6)	5 (11,6)	2 (11,8)	2 (6,9)		21 (15,4)	6 (10,7)	3 (8,8)	4 (13,3)	1 (3,6)
	Fehlend	*35*						*157*						*165*						*163*					
			p n.s.; Cramer's V: ,078						p n.s.; Cramer's V: ,103						p n.s.; Cramer's V: ,094						p n.s.; Cramer's V: ,117				
**KV-Region[2]**			**(N=34)**	**(N=48)**	**(N=61)**	**(N=74)**	**(N=198)**		**(N=35)**	**(N=24)**	**(N=60)**	**(N=86)**	**(N=88)**		**(N=129)**	**(N=67)**	**(N=43)**	**(N=17)**	**(N=29)**		**(N=138)**	**(N=57)**	**(N=35)**	**(N=29)**	**(N=28)**
	Region West		11 (32,4)	20 (41,7)	22 (36,1)	23 (31,1)	68 (34,3)		6 (17,1)	8 (33,3)	19 (31,7)	32 (37,2)	31 (35,2)		33 (25,6)	24 (35,8)	16 (37,2)	7 (41,2)	14 (48,3)		43 (31,2)	20 (35,1)	14 (40,0)	5 (17,2)	12 (42,9)
	Region Nord		4 (11,8)	1 (2,1)	7 (11,5)	7 (9,5)	35 (17,7)		10 (28,6)	1 (4,2)	7 (11,7)	7 (8,1)	5 (5,7)		18 (14,0)	4 (6,0)	5 (11,6)	1 (5,9)	2 (6,9)		20 (14,5)	4 (7,0)	3 (8,6)	1 (3,4)	2 (7,1)
	Region Süd		4 (11,8)	10 (20,8)	16 (26,2)	24 (32,4)	66 (33,3)		10 (28,6)	8 (33,3)	22 (36,7)	28 (32,6)	17 (19,3)		45 (34,9)	21 (31,3)	10 (23,3)	3 (17,6)	4 (13,8)		32 (23,2)	17 (29,8)	11 (31,4)	17 (58,6)	8 (28,6)
	Region Ost		3 (8,8)	10 (20,8)	4 (6,6)	6 (8,1)	17 (8,6)		4 (11,4)	3 (12,5)	3 (5,0)	10 (11,6)	12 (13,6)		12 (9,3)	10 (14,9)	3 (7,0)	2 (11,8)	3 (10,3)		16 (11,6)	9 (15,8)	2 (5,7)	1 (3,4)	1 (3,6)
	Berlin		6 (17,6)	4 (8,3)	7 (11,5)	10 (13,5)	6 (3,0)		2 (5,7)	4 (16,7)	5 (8,3)	5 (5,8)	13 (14,8)		8 (6,2)	6 (9,0)	5 (11,6)	3 (17,6)	5 (17,2)		14 (10,1)	4 (7,0)	2 (5,7)	3 (10,3)	5 (17,9)
	Hamburg		6 (17,6)	3 (6,3)	5 (8,2)	4 (5,4)	6 (3,0)		3 (8,6)	0 (0,0)	4 (6,7)	4 (4,7)	10 (11,4)		13 (10,1)	2 (3,0)	4 (9,3)	1 (5,9)	1 (3,4)		13 (9,4)	3 (5,3)	3 (8,6)	2 (6,9)	0 (0,0)
	Fehlend	*32*						*154*						*162*						*160*					
			p <0,001; Cramer's V: ,171						p <0,05; Cramer's V: ,174						p n.s.; Cramer's V: ,146						p n.s.; Cramer's V: ,162				
**Gemeindegröße**			**(N=34)**	**(N=49)**	**(N=60)**	**(N=74)**	**(N=197)**		**(N=35)**	**(N=23)**	**(N=60)**	**(N=87)**	**(N=88)**		**(N=129)**	**(N=66)**	**(N=43)**	**(N=18)**	**(N=29)**		**(N=138)**	**(N=56)**	**(N=35)**	**(N=30)**	**(N=28)**

	Landgemeinde	2 (5,9)	5 (10,2)	2 (3,3)	5 (6,8)	21 (10,7)	2 (5,7)	1 (4,3)	4 (6,7)	7 (8,0)	3 (3,4)	10 (7,8)	3 (4,5)	1 (2,3)	1 (5,6)	2 (6,9)	10 (7,2)	3 (5,4)	1 (2,9)	2 (6,7)	3 (10,7)
	Kleinstadt	3 (8,8)	9 (18,4)	9 (15,0)	10 (13,5)	35 (17,8)	7 (20,0)	2 (8,7)	8 (13,3)	12 (13,8)	12 (13,6)	24 (18,6)	6 (9,1)	4 (9,3)	2 (11,1)	4 (13,8)	19 (13,8)	4 (7,1)	7 (20,0)	6 (20,0)	4 (14,3)
	Mittelstadt	9 (26,5)	8 (16,3)	13 (21,7)	19 (25,7)	52 (26,4)	11 (31,4)	5 (21,7)	11 (18,3)	24 (27,6)	21 (23,9)	31 (24,0)	17 (25,8)	6 (14,0)	5 (27,8)	11 (37,9)	29 (21,0)	16 (28,6)	8 (22,9)	7 (23,3)	11 (39,3)
	Großstadt	20 (58,8)	27 (55,1)	36 (60,0)	40 (54,1)	89 (45,2)	15 (42,9)	15 (65,2)	37 (61,7)	44 (50,6)	52 (59,1)	64 (49,6)	40 (60,6)	32 (74,4)	10 (55,6)	12 (41,4)	80 (58,0)	33 (58,9)	19 (54,3)	15 (50,0)	10 (35,7)
	Fehlend	*33*					*154*					*162*					*160*				
		p n.s.; Cramer's V: ,092					p n.s.; Cramer's V: ,093					p n.s.; Cramer's V: ,132					p n.s.; Cramer's V: ,114				
**Patienten-gruppe[3]**		**(N=33)**	**(N=48)**	**(N=60)**	**(N=74)**	**(N=196)**	**(N=33)**	**(N=24)**	**(N=59)**	**(N=86)**	**(N=88)**	**(N=127)**	**(N=66)**	**(N=43)**	**(N=17)**	**(N=29)**	**(N=136)**	**(N=56)**	**(N=34)**	**(N=30)**	**(N=28)**
	Ausschließlich Erwachsene	30 (90,9)	44 (91,7)	52 (86,7)	64 (86,5)	177 (90,3)	27 (81,8)	21 (87,5)	52 (88,1)	81 (94,2)	80 (90,9)	116 (91,3)	60 (90,9)	39 (90,7)	15 (88,2)	23 (79,3)	124 (91,2)	50 (89,3)	30 (88,2)	27 (90,0)	24 (85,7)
	Erwachsene/Kinder/Jugendliche	3 (9,1)	4 (8,3)	8 (13,3)	10 (13,5)	19 (9,7)	6 (18,2)	3 (12,5)	7 (11,9)	5 (5,8)	8 (9,1)	11 (8,7)	6 (9,1)	4 (9,3)	2 (11,8)	6 (20,7)	12 (8,8)	6 (10,7)	4 (11,8)	3 (10,0)	4 (14,3)
	Fehlend	*36*					*157*					*165*					*163*				
		p n.s.; Phi: ,063					p n.s.; Phi: ,126					p n.s.; Phi: ,118					p n.s.; Phi: ,056				

[1]Aufgrund geringer Fallzahlen wurden die Altersgruppen 21-30 und 31-40 zusammengefasst.
[2]KV-Region West (Westfalen-Lippe, Nordrhein, Hessen, Rheinland-Pfalz, Saarland), KV-Region Nord (Schleswig-Holstein, Niedersachsen, Bremen), KV-Region Süd (Baden-Württemberg, Bayern), KV-Region Ost (Mecklenburg-Vorpommern, Brandenburg, Sachsen, Sachsen-Anhalt, Thüringen).
[3]Wenn beide Gruppen mindestens einen Anteil von ca. einem Drittel der betreuten Patienten ausmacht.

Anhang 6: Subgruppenanalysen zur Beurteilung der Zusammenarbeit mit der TSS stratifiziert nach den Merkmalen der Psychotherapeuten

**Zufriedenheit mit …**		**der Zusammenarbeit insgesamt.**					**den Informationen, die die TSS an Patienten vermittelt.**					**der Terminvergabe.**					**dem zeitlichen Aufwand, um Termine zu melden.**				
		**Sehr un-zufrieden N (%)**	**Eher un-zufrieden N (%)**	**Teils, teils N (%)**	**Eher zufrieden N (%)**	**Sehr zufrieden N (%)**	**Sehr un-zufrieden N (%)**	**Eher un-zufrieden N (%)**	**Teils, teils N (%)**	**Eher zufrieden N (%)**	**Sehr zufrieden N (%)**	**Sehr un-zufrieden N (%)**	**Eher un-zufrieden N (%)**	**Teils, teils N (%)**	**Eher zufrieden N (%)**	**Sehr zufrieden N (%)**	**Sehr un-zufrieden N (%)**	**Eher un-zufrieden N (%)**	**Teils, teils N (%)**	**Eher zufrieden N (%)**	**Sehr zufrieden N (%)**
**Alter[1]**		**(N=21)**	**(N=43)**	**(N=69)**	**(N=106)**	**(N=30)**	**(N=32)**	**(N=73)**	**(N=87)**	**(N=55)**	**(N=17)**	**(N=21)**	**(N=36)**	**(N=58)**	**(N=111)**	**(N=38)**	**(N=38)**	**(N=59)**	**(N=40)**	**(N=93)**	**(N=39)**
	21 - 40	4 (19,0)	3 (7,0)	10 (14,5)	10 (9,4)	2 (6,7)	3 (9,4)	11 (15,1)	10 (11,5)	4 (7,3)	1 (5,9)	0 (0,0)	8 (22,2)	5 (8,6)	14 (12,6)	2 (5,3)	3 (7,9)	5 (8,5)	4 (10,0)	11 (11,8)	6 (15,4)
	41 - 50	3 (14,3)	14 (32,6)	18 (26,1)	23 (21,7)	9 (30,0)	10 (31,3)	19 (26,0)	20 (23,0)	13 (23,6)	5 (29,4)	5 (23,8)	10 (27,8)	16 (27,6)	27 (24,3)	9 (23,7)	13 (34,2)	19 (32,2)	5 (12,5)	21 (22,6)	11 (28,2)
	51 - 60	4 (19,0)	14 (32,6)	20 (29,0)	45 (42,5)	12 (40,0)	10 (31,3)	22 (30,1)	32 (36,8)	20 (36,4)	9 (52,9)	7 (33,3)	9 (25,0)	16 (27,6)	41 (36,9)	19 (50,0)	6 (15,8)	21 (35,6)	17 (42,5)	33 (35,5)	17 (43,6)
	über 60	10 (47,6)	12 (27,9)	21 (30,4)	28 (26,4)	7 (23,3)	9 (28,1)	21 (28,8)	25 (28,7)	18 (32,7)	2 (11,8)	9 (42,9)	9 (25,0)	21 (36,2)	29 (26,1)	8 (21,1)	16 (42,1)	14 (23,7)	14 (35,0)	28 (30,1)	5 (12,8)
	Fehlend	*178*					*183*					*183*					*178*				
		p n.s.; Cramer's V: ,129					p n.s.; Cramer's V: ,097					p n.s.; Cramer's V: ,146					p n.s.; Cramer's V: ,154				
**Geschlecht**		**(N=19)**	**(N=42)**	**(N=68)**	**(N=101)**	**(N=29)**	**(N=30)**	**(N=71)**	**(N=83)**	**(N=53)**	**(N=17)**	**(N=20)**	**(N=34)**	**(N=55)**	**(N=109)**	**(N=36)**	**(N=37)**	**(N=55)**	**(N=39)**	**(N=90)**	**(N=38)**
	Männlich	4 (21,1)	11 (26,2)	13 (19,1)	20 (19,8)	10 (34,5)	6 (20,0)	15 (21,1)	18 (21,7)	10 (18,9)	7 (41,2)	3 (15,0)	9 (26,5)	13 (23,6)	22 (20,2)	10 (27,8)	9 (24,3)	13 (23,6)	8 (20,5)	17 (18,9)	11 (28,9)
	Weiblich	15 (78,9)	31 (73,8)	55 (80,9)	81 (80,2)	19 (65,5)	24 (80,0)	56 (78,9)	65 (78,3)	43 (81,1)	10 (58,8)	17 (85,0)	25 (73,5)	42 (76,4)	87 (79,8)	26 (72,2)	28 (75,7)	42 (76,4)	31 (79,5)	73 (81,1)	27 (71,1)
	Fehlend	*188*					*193*					*193*					*188*				
		p n.s.; Phi: ,118					p n.s.; Phi: ,126					p n.s.; Phi: ,087					p n.s.; Phi: ,083				
**Studiengang**		**(N=20)**	**(N=42)**	**(N=70)**	**(N=104)**	**(N=29)**	**(N=30)**	**(N=72)**	**(N=87)**	**(N=55)**	**(N=16)**	**(N=20)**	**(N=37)**	**(N=56)**	**(N=110)**	**(N=37)**	**(N=37)**	**(N=59)**	**(N=40)**	**(N=92)**	**(N=37)**
	Medizin	1 (5,0)	4 (9,5)	8 (11,4)	12 (11,5)	3 (10,3)	2 (6,7)	5 (6,9)	12 (13,8)	7 (12,7)	2 (12,5)	3 (15,0)	3 (8,1)	5 (8,9)	16 (14,5)	1 (2,7)	4 (10,8)	7 (11,9)	5 (12,5)	6 (6,5)	6 (16,2)
	Psychologie	18 (90,0)	35 (83,3)	62 (88,6)	90 (86,5)	26 (89,7)	26 (86,7)	64 (88,9)	75 (86,2)	47 (85,5)	14 (87,5)	16 (80,0)	32 (86,5)	51 (91,1)	92 (83,6)	35 (94,6)	33 (89,2)	50 (84,7)	35 (87,5)	82 (89,1)	31 (83,8)
	Anderes Studium	1 (5,0)	3 (7,1)	0 (0,0)	2 (1,9)	0 (0,0)	2 (6,7)	3 (4,2)	0 (0,0)	1 (1,8)	0 (0,0)	1 (5,0)	2 (5,4)	0 (0,0)	2 (1,8)	1 (2,7)	0 (0,0)	2 (3,4)	0 (0,0)	4 (4,3)	0 (0,0)
	Fehlend	*182*					*187*					*187*					*182*				
		p n.s.; Cramer's V: ,125					p n.s.; Cramer's V: ,128					p n.s.; Cramer's V: ,129					p n.s.; Cramer's V: ,120				
**PT-Verfahren**		**(N=20)**	**(N=43)**	**(N=70)**	**(N=106)**	**(N=30)**	**(N=30)**	**(N=74)**	**(N=87)**	**(N=56)**	**(N=17)**	**(N=20)**	**(N=37)**	**(N=57)**	**(N=112)**	**(N=38)**	**(N=37)**	**(N=60)**	**(N=40)**	**(N=94)**	**(N=38)**
	Nur Tiefenpsychologisch	4 (20,0)	11 (25,6)	19 (27,1)	29 (27,4)	8 (26,7)	7 (23,3)	16 (21,6)	25 (28,7)	21 (37,5)	2 (11,8)	6 (30,0)	11 (29,7)	13 (22,8)	35 (31,3)	6 (15,8)	9 (24,3)	14 (23,3)	12 (30,0)	30 (31,9)	7 (18,4)
	Nur Verhaltenstherapie	12 (60,0)	28 (65,1)	39 (55,7)	53 (50,0)	16 (53,3)	19 (63,3)	45 (60,8)	44 (50,6)	25 (44,6)	11 (64,7)	9 (45,0)	22 (59,5)	31 (54,4)	59 (52,7)	23 (60,5)	21 (56,8)	36 (60,0)	17 (42,5)	50 (53,2)	24 (63,2)
	Nur Analytisch und Tiefenpsychologisch	1 (5,0)	2 (4,7)	8 (11,4)	19 (17,9)	4 (13,3)	1 (3,3)	8 (10,8)	13 (14,9)	9 (16,1)	2 (11,8)	2 (10,0)	4 (10,8)	7 (12,3)	15 (13,4)	5 (13,2)	3 (8,1)	5 (8,3)	9 (22,5)	10 (10,6)	6 (15,8)
	Fehlend	*178*					*183*					*183*					*178*				
		p n.s.; Cramer's V: ,114					p n.s.; Cramer's V: ,133					p n.s.; Cramer's V: ,134					p n.s.; Cramer's V: ,126				
**KV-Region[2]**		**(N=21)**	**(N=44)**	**(N=70)**	**(N=107)**	**(N=30)**	**(N=32)**	**(N=74)**	**(N=88)**	**(N=56)**	**(N=17)**	**(N=21)**	**(N=37)**	**(N=58)**	**(N=113)**	**(N=38)**	**(N=38)**	**(N=60)**	**(N=40)**	**(N=95)**	**(N=39)**
	Region West	6 (28,6)	19 (43,2)	25 (35,7)	36 (33,6)	10 (33,3)	14 (43,8)	24 (32,4)	30 (34,1)	19 (33,9)	6 (35,3)	3 (14,3)	17 (45,9)	19 (32,8)	44 (38,9)	10 (26,3)	13 (34,2)	18 (30,0)	8 (20,0)	39 (41,1)	15 (38,5)
	Region Nord	5 (23,8)	4 (9,1)	7 (10,0)	10 (9,3)	0 (0,0)	5 (15,6)	9 (12,2)	8 (9,1)	5 (8,9)	0 (0,0)	5 (23,8)	2 (5,4)	2 (3,4)	14 (12,4)	3 (7,9)	5 (13,2)	6 (10,0)	4 (10,0)	8 (8,4)	4 (10,3)
	Region Süd	7 (33,3)	14 (31,8)	19 (27,1)	26 (24,3)	8 (26,7)	9 (28,1)	16 (21,6)	27 (30,7)	16 (28,6)	3 (17,6)	10 (47,6)	10 (27,0)	20 (34,5)	24 (21,2)	10 (26,3)	13 (34,2)	25 (41,7)	15 (37,5)	19 (20,0)	4 (10,3)
	Region Ost	2 (9,5)	3 (6,8)	8 (11,4)	10 (9,3)	4 (13,3)	2 (6,3)	10 (13,5)	7 (8,0)	6 (10,7)	2 (11,8)	1 (4,8)	2 (5,4)	6 (10,3)	13 (11,5)	4 (10,5)	3 (7,9)	4 (6,7)	6 (15,0)	9 (9,5)	5 (12,8)
	Berlin	1 (4,8)	2 (4,5)	4 (5,7)	15 (14,0)	6 (20,0)	2 (6,3)	7 (9,5)	8 (9,1)	6 (10,7)	5 (29,4)	1 (4,8)	5 (13,5)	4 (6,9)	9 (8,0)	8 (21,1)	3 (7,9)	3 (5,0)	2 (5,0)	11 (11,6)	9 (23,1)

	Hamburg	0 (0,0)	2 (4,5)	7 (10,0)	10 (9,3)	2 (6,7)	0 (0,0)	8 (10,8)	8 (9,1)	4 (7,1)	1 (5,9)	1 (4,8)	1 (2,7)	7 (12,1)	9 (8,0)	3 (7,9)	1 (2,6)	4 (6,7)	5 (12,5)	9 (9,5)	2 (5,1)
	Fehlend	*175*					*180*					*180*					*175*				
		p n.s.; Cramer's V: ,140					p n.s.; Cramer's V: ,129					p n.s.; Cramer's V: ,169					p <0,05; Cramer's V: ,171				
**Gemeindegröße**		**(N=21)**	**(N=44)**	**(N=70)**	**(N=106)**	**(N=30)**	**(N=32)**	**(N=74)**	**(N=88)**	**(N=56)**	**(N=17)**	**(N=21)**	**(N=37)**	**(N=58)**	**(N=113)**	**(N=38)**	**(N=38)**	**(N=60)**	**(N=40)**	**(N=95)**	**(N=39)**
	Landgemeinde	0 (0,0)	4 (9,1)	4 (5,7)	5 (4,7)	3 (10,0)	2 (6,3)	7 (9,5)	5 (5,7)	2 (3,6)	0 (0,0)	1 (4,8)	2 (5,4)	5 (8,6)	5 (4,4)	2 (5,3)	1 (2,6)	5 (8,3)	3 (7,5)	5 (5,3)	2 (5,1)
	Kleinstadt	4 (19,0)	8 (18,2)	6 (8,6)	13 (12,3)	4 (13,3)	6 (18,8)	9 (12,2)	9 (10,2)	11 (19,6)	0 (0,0)	4 (19,0)	5 (13,5)	8 (13,8)	15 (13,3)	3 (7,9)	5 (13,2)	13 (21,7)	5 (12,5)	11 (11,6)	1 (2,6)
	Mittelstadt	7 (33,3)	11 (25,0)	17 (24,3)	20 (18,9)	8 (26,7)	8 (25,0)	12 (16,2)	24 (27,3)	13 (23,3)	5 (29,4)	7 (33,3)	10 (27,0)	12 (20,7)	26 (23,0)	8 (21,1)	15 (39,5)	12 (20,0)	9 (22,5)	21 (22,1)	8 (20,5)
	Großstadt	10 (47,6)	21 (47,7)	43 (61,4)	68 (64,2)	15 (50,0)	16 (50,0)	46 (62,2)	50 (56,8)	30 (53,6)	12 (70,6)	9 (42,9)	20 (54,1)	33 (56,9)	67 (59,3)	25 (65,8)	17 (44,7)	30 (50,0)	23 (57,5)	58 (61,1)	28 (71,8)
	Fehlend	*176*					*180*					*180*					*175*				
		p n.s.; Cramer's V: ,112					p n.s.; Cramer's V: ,124					p n.s.; Cramer's V: ,082					p n.s.; Cramer's V: ,142				
**Patienten-gruppe**[3]		**(N=20)**	**(N=43)**	**(N=70)**	**(N=106)**	**(N=30)**	**(N=30)**	**(N=74)**	**(N=87)**	**(N=56)**	**(N=17)**	**(N=20)**	**(N=37)**	**(N=57)**	**(N=112)**	**(N=38)**	**(N=37)**	**(N=60)**	**(N=40)**	**(N=94)**	**(N=38)**
	Ausschließlich Erwachsene	19 (95,0)	34 (79,1)	66 (94,3)	96 (90,6)	25 (83,3)	28 (93,3)	60 (81,1)	83 (95,4)	49 (87,5)	15 (88,2)	19 (95,0)	29 (78,4)	48 (84,2)	106 (94,6)	33 (86,8)	31 (83,8)	54 (90,0)	35 (87,5)	86 (91,5)	34 (89,5)
	Erwachsene/Kinder/ Jugendliche	1 (5,0)	9 (20,9)	4 (5,7)	10 (9,4)	5 (16,7)	2 (6,7)	14 (18,9)	4 (4,6)	7 (12,5)	2 (11,8)	1 (5,0)	8 (21,6)	9 (15,8)	6 (5,4)	5 (13,2)	6 (16,2)	6 (10,0)	5 (12,5)	8 (8,5)	4 (10,5)
	Fehlend	*178*					*183*					*183*					*178*				
		p n.s.; Phi: ,177					p n.s.; Phi: ,186					p <0,05; Phi: ,196					p n.s.; Phi: ,082				

[1]Aufgrund geringer Fallzahlen wurden die Altersgruppen 21-30 und 31-40 zusammengefasst.

[2]KV-Region West (Westfalen-Lippe, Nordrhein, Hessen, Rheinland-Pfalz, Saarland), KV-Region Nord (Schleswig-Holstein, Niedersachsen, Bremen), KV-Region Süd (Baden-Württemberg, Bayern), KV-Region Ost (Mecklenburg-Vorpommern, Brandenburg, Sachsen, Sachsen-Anhalt, Thüringen).

[3]Wenn beide Gruppen mindestens einen Anteil von ca. einem Drittel der betreuten Patienten ausmacht.
